# A nationwide cross-sectional study of workers’ mental health during the COVID-19 pandemic: Impact of changes in working conditions, financial hardships, psychological detachment from work and work-family interface

**DOI:** 10.1186/s40359-022-00783-y

**Published:** 2022-03-18

**Authors:** Mario Alberto Trógolo, Luciana Sofía Moretti, Leonardo Adrián Medrano

**Affiliations:** 1 Universidad Siglo 21, Bv. de los Latinos 8555, 5000 Córdoba, Argentina; 2 Pontifica Universidad Católica Madre y Maestra, Santiago De Los Caballeros, República Dominicana

**Keywords:** COVID-19, Telework, Unemployment, Income loss, Financial problems, Mental health

## Abstract

**Background:**

The COVID-19 disease has changed people’s work and income. While recent evidence has documented the adverse impact of these changes on mental health outcomes, most research is focused on frontline healthcare workers and the reported association between income loss and mental health comes from high-income countries. In this study we examine the impact of changes in working conditions and income loss related to the COVID-19 lockdown on workers’ mental health in Argentina. We also explore the role of psychological detachment from work and work-family interaction in mental health.

**Methods:**

A total of 1049 participants aged between 18 and 65 who were working before the national lockdown in March 2020 were recruited using a national random telephone survey. Work conditions included: working at the usual workplace during the pandemic, working from home with flexible or fixed schedules, and being unemployed or unable to work due to the pandemic. Measures of financial hardship included income loss and self-reported financial problems related to the outbreak. Work-family interface included measures of work-family conflict (WFC) and family-work conflict (FWC). Mental health outcomes included burnout, life satisfaction, anxiety and depressive symptoms. Data were collected in October 2020.

**Results:**

Home-based telework under fixed schedules and unemployment impact negatively on mental health. Income loss and particularly self-reported financial problems were also associated with deterioration of mental health. More than half of the participants reported financial problems, and those who became unemployed during the pandemic experienced more often financial problems. Finally, psychological detachment from work positively influenced mental health; WFC and FWC were found to negatively impact on mental health.

**Conclusions:**

Countries’ policies should focus on supporting workers facing economic hardships and unemployment to ameliorate the COVID-19’ negative impact on mental health. Organisations can protect employees’ mental health by actively encouraging psychological detachment from work and by help managing work-family interface. Longitudinal studies are needed to more thoroughly assess the long-term impact of the COVID-19-related changes in work and economic turndown on mental health issues.

## Background

A new highly infectious coronavirus known as SARS-COV-2 or COVID-19 appeared in December 2019 in Wuhan, China, and rapidly disseminated worldwide [1], being officially declared a global pandemic in March 2020 [2]. Most countries have adopted different strategies to contain the spread of the virus—not only imported cases, but also local transmission [3]. Key measures imposed by governments included social distancing and lockdown implying the obligation to stay at home, which has had a profound impact on people’s work and lives. Regarding life, social relations and even contact with close family members have been severely restricted or completely absent for several months. Regarding work, many organisations have mandated employees to work from home; some have reduced or shut down their production due to the economic breakdown, and others have closed down, particularly contact-sensitive sectors—e.g., hotels, bars, restaurants, shops—that were hit the hardest by the lockdown [4, 5]. As a result, some workers have lost their jobs, others have become inactive due to the impossibility of performing regular work activities from home and in many jobs people were forced to home-based telework. In parallel to changes in working conditions, many workers suffered income losses and companies from different sectors have downsized salaries due to decline in production and the economic crisis [6]. These changes in working conditions and the financial insecurity coupled with uncertainty about the course of the pandemic are likely to have adverse effects on workers’ mental health.

The COVID-19 lockdown has not only affected work, but also family life and their interaction [5]. In this regard, the boundaries between work and family domains have become blurred, particularly for workers who shifted to working from home [7]. Past research has shown that working from home has potential benefits, such as more flexibility to structure workday and balance home and work demands [8]; however, it has also been revealed a greater risk that work spill over into home [9]. Thus, people working from home may experience conflict between different roles, in the form of works demands negatively interfering with family duties (i.e., work-family conflict; WFC). In addition, staying 24/7 at home during the lockdown is likely to increase demands experienced at home (e.g., childcare and family demands) which may negatively interfere with work demands, leading to family-work conflict (FWC). According to the scarcity hypothesis, family and work domains compete for limited time- and energy-related resources which in turn negatively affect workers’ work-life balance [10]. Moreover, the lack of differentiated physical spaces between work and home may hinder psychological detachment from work—i.e., stop thinking about job-related matters and working during non-work time [11]. A recent study [12] showed that people working from home during the pandemic spend more time in work roles than in family roles, or in a combination of both, suggesting that they have difficulties to transition from work to family roles and disconnect from work, thus impeding recovery and well-being.

In addition to people working from home, it is also possible that work-family interface and psychological detachment from work are affected across different working conditions, including workers who are unable to work or unemployed as a result of COVID-19 lockdown. Investigating these issues during unemployment may appear contradictory. It could be reasonable to argue that because there is no paid work, neither work-family interface nor psychological detachment from work exist. Nevertheless, work is an integral part of people’s lives, even during unemployment [13]. For example, unemployed individuals may invest a significant amount of time and energy in seeking for a new job while being at home, or be continuously self-absorbed by persistent rumination about (lack of) work, which may reduce their available resources to respond to family demands at home. Accordingly, work-family interface and psychological detachment from work also exist during unemployment, but our knowledge about this and their impact on well-being and mental health is limited.

This study examines the impact of changes in working conditions, financial hardships, work-family interface and psychological detachment from work on workers’ mental health during the COVID-19 pandemic. Although the effects of these variables on employees’ well-being and mental health have been extensively studied [14–19], these studies were conducted under vastly different and less extreme circumstances than the current situation. Thus, findings may be hard to transfer due to the unprecedented and unique characteristics of the pandemic. Accordingly, the aim of this study is to evaluate the impact of the changes caused by the COVID-19 lockdown on several indicators of workers’ mental health, including anxiety, depression, burnout, and life satisfaction. Specifically, we analyze: (a) the impact of working conditions, including work in the usual workplace, work from home with fixed or flexible schedules, and unemployment; (b) the impact of financial hardships—income loss and self-reported financial problems—; (c) the impact of WFC and FWC; and (d) the influence of psychological detachment from work.

The current study contributes to the growing body of research on COVID-19 mental health outcomes for workers in at least three ways. Firstly, empirical research has been primarily focused on frontline healthcare workers [20–28]. Evidence of the impact of the COVID-19 on non-healthcare workers is scant. Xiao et al. [29] reported decreased physical and mental well-being in a sample of Chinese workers following the transition to work from home. Evanoff et al. [30] found a high prevalence of stress, anxiety, depression and burnout, and worsened well-being among university employees and postdoctoral fellows after 4–5 weeks of work-from-home plans enacted by the university. Hwang et al. [31] found increased levels of burnout in a sample of South Korean employees from various service sectors after the onset of the COVID-19 pandemic; however, they did not discriminate among employees who worked from home, those who worked at their workplace, and those who did not work due to the lockdown. In short, objective data on mental health outcomes of the COVID-19 pandemic in workers outside the healthcare sector are scarce and limited to home-based telework (see also [12, 32, 33]). Secondly, studies addressing mental health issues of income loss in workers during the COVID-19 pandemic focus on high-income countries, such as China [34], Thailand [35], USA [36] and Europe [37]. To the best of our knowledge, this is the first study to examine the impact of income loss on the mental health of workers in a low-to-middle income Western country. Lastly, research assessing the impact of COVID-19 on the mental health of workers was typically conducted at the onset or after the first weeks of the pandemic and, as such, only reflects short-term effects; we provide new evidence of the long-term influence of working conditions and income loss on workers’ mental health by examining these issues several months after the beginning of mandatory strict lockdown policies imposed by the Argentine’ government.

## Methods

### Participants

A sample of 1049 argentine workers was recruited using a national random telephone survey. First, different geographic areas and the corresponding phone codes were identified. Then, the telephone numbers to be dialed were randomly selected. Household residents were eligible if they met the following criteria: (1) aged between 18 and 65 years old, and (2) working before to the national lockdown policies in response to COVID-19 disease. Fifty-one percent of the respondents were male. The mean age was 42.15 (SD = 12.61). The majority of the participants (44.5%) held a university or postgraduate educational-level, 28% held a secondary educational-level, 22.5% held a tertiary educational-level, and the remaining participants (5%) held a primary educational-level. With regard to working conditions during COVID-19 lockdown, 27.9% of the respondents continued to work at their usual workplace, 19.8% worked from home with a flexible schedule, 13.6% also worked from home although with fixed schedules, and 38.7% were not working or become unemployed. With respect to income loss, 48.3% percent of the workers reported no monthly income loss since the lockdown and, among those who did, 6.2% reported income loss of less than 20%, 10.4% reported income loss between 21 and 40%, 13% reported income loss between 41 and 60%, 7.2% reported income loss between 61 and 80%, and 14.9% reported income loss of 80% or more. Finally, 56.1% of the respondents reported financial problems.

### Measures

#### Independent variables

The independent variables were changes in working conditions, financial hardship, psychological detachment from work, WFC and FWC related to the COVID‐19 outbreak. Change in working conditions was assessed as follow: continue working in the usual workplace, working from home with fixed schedule (e.g., 9 am to 5 pm), working from home with flexible schedule and unable to work or unemployed. Financial hardship included measures of objective income loss and the perception of financial strain. Income loss was assessed by asking people how their incomes has changed relative to before the pandemic; responses were categorized as no, less than 20%, between 21 and 40%, between 41 and 60%, between 61 and 80%, and 80% or more of monthly income. Self-reported financial problems (e.g., indebtedness, financial shortage ranging from difficulty in paying the rent to paying at the supermarket, etc.) were assessed using a dichotomous question (yes/no). The Recovery Experience Questionnaire (REQ, [38]) and the Survey Work-Home Interaction—Nijmegen (SWING, [39]) were used to assess psychological detachment from work and work-family interface, respectively.

The REQ is a 16-item scale assessing four experiences associated to individuals’ unwinding and recuperation from work during leisure time: psychological detachment from work, relaxation, mastery, and control. In the present study, we used the four items that correspond to the psychological detachment from work subscale. An example of item is “During after-work hours, I forget about work”. All items are rated on a 5-point Likert scale ranging from 1 (I fully disagree) to 5 (I fully agree). In this study, we used the Argentinian validation of the REQ [40] which consists of 16 items, as in the original scale. Confirmatory factor analysis supported the original four-factor structure. All the factors showed good internal consistency (Cronbach’s alpha [α] ranging from 0.75 to 0.92) and theoretically significant associations between the REQ, on the one hand, and measures of work engagement, burnout and affect, on the other supported for concurrent validity. In the current sample, Cronbach’s α for the psychological detachment from work subscale is 0.72.

The SWING consists of 22 items tapping four types of work-home interaction: positive work-home interaction, positive home-work interaction, negative work-home interaction, and negative home-work interaction. In this study we used the latter two subscales to assess WFC and FWC, respectively. Examples of items are “How often does it happen that you find it difficult to fulfill your domestic obligations because you are constantly thinking about your work?” (WFC), and “How often does it happen that you have difficulty concentrating on your work because you are preoccupied with domestic matters?” (FWC). Participants should indicate how often their experienced the interactions between work and home using a 4-point Likert type scale ranging from 0 (never) to 3 (always). A recent study in Argentina [41] supported the factorial validity of the SWING using exploratory and confirmatory factor analysis. Cronbach’s alpha coefficients ranged between 0.74 and 0.76 for the subscales, indicating acceptable levels of internal consistency. In the present study, Cronbach’s α for WFC and FWC are 0.90 and 0.89, respectively.

#### Mental health outcomes

The mental health outcomes were assessed with the Maslach Burnout Inventory-General Survey (MBI-GS, [42]), the Patient Health Questionnaire—9‐items (PHQ-9, [43]), the Generalised Anxiety Disorder Scale—7‐items (GAD, [44]), and the Satisfaction with Life Scale (SWLS, [45]).

The MBI-GS is the most widely used measure for the assessment of burnout. The original scale contains 16 items tapping three dimensions of burnout: emotional exhaustion, cynicism and reduced professional self-efficacy [42]. However, emotional exhaustion and cynicism are considered the core burnout dimensions, while there is disagreement regarding the role of professional self-efficacy as a constituent part of the burnout syndrome [46–48]. Consistent with this, studies conducted in Argentina [49] did not support the original MBI-GS thee-factor structure; instead, a two-factor model consisting of the “core” burnout fit the data well. Reliability analysis (Cronbach’s α) showed good internal consistency for the exhaustion and cynicism scales (0.73 and 0.78, respectively), and significant and theoretically expected associations with measures of burnout and affect provided support for concurrent validity of the scale. Accordingly, in the present study we assessed emotional exhaustion and cynicism. The scale items are rated on a 7-point frequency scale ranging from 0 (never) to 6 (daily). In the current study, Cronbach’s α for exhaustion and cynicism are 0.80 and 0.83, respectively.

The PHQ‐9 is a one-dimensional scale consisting of nine items that assess the presence of the nine diagnostic criteria for depression according to DSM-IV. The PHQ-9 evaluates the presence of the following symptoms over the previous two-week period: (a) depressed mood; (b) anhedonia; (c) sleep problems; (d) feelings of tiredness; (e) changes in appetite or weight; (f) feelings of guilt or worthlessness; (g) difficulty concentrating; (h) feelings of sluggishness or worry; (i) suicidal ideation. Items are answered on a 4-point Likert scale from 0 to 3 as follows: 0 (never), 1 (several days), 2 (more than half of the days), and 3 (most days). In Argentina, confirmatory factor analysis supported the unidimensionality of the PHQ-9. Internal consistency was satisfactory (McDonald’s ω = 0.89), and sensitivity and specificity tests supported the utility and accuracy of the PHQ-9 as a screening tool for diagnosing major depression [50]. In the present study, the total scale score was used; the higher the score, the higher the presence of depressive symptoms. The Cronbach’s α for the scale in the current sample is 0.83.

The GAD‐7 consists of seven items assessing anxiety symptoms described in the DSM-IV: (a) jitters; (b) excessive restlessness; (c) fatigue; (d) muscular pain or tension; (e) sleeping problems; (f) attention problems and (g) easy irritability. Participants were asked to indicate the extent to which they experienced each symptom within the past two weeks, using a 4-point Likert-type scale ranging from 0 (never) to 1 (several days), 2 (more than half the days) and 3 (nearly every day). The examination of psychometric properties of the GAD-7 in Argentina [51] revealed a one-dimensional factor structure, consistent with the original scale. Reliability analyses showed satisfactory indexes (Cronbach’s α = 83; McDonald’s ω = 0.84) and sensitivity and specificity analyses supported the utility of GAD-7 for detecting general anxiety disorder. In the present study, the total scale score was used; the higher the score, the higher the presence of anxiety symptoms. In the current sample, Cronbach’s α for the GAD-7 is 0.86.

The SWLS consists of 5 items assessing overall life satisfaction. Items are answered on a 7-point Likert scale, ranging from 1 (strongly disagree) to 7 (strongly agree). A study conducted by Moyano et al. [52] indicated satisfactory evidence of validity and reliability of the SWLS in Argentina. In particular, exploratory factor analysis yielded a one-factor solution, consistent with the original SWLS. The internal consistency (Cronbach’s α) for the scale was 0.76, and convergent validity of the SWLS was supported by the significant associations with measures of job satisfaction and psychological well-being. The Cronbach’s α coefficient for the SWLS in the present study is 0.79.

Socio-demographic questionnaire*.* Participants’ information was obtained about sex, age, educational background, job status, occupational sector and type of company.

### Procedure and data analysis

Data were collected by four experienced telephone interviewers using random-digit-dialing methodology**.** Subjects were invited to participate in a national survey on work during the COVID-19 pandemic. The response rates to phone calls were high (96%). Data collection was carried out in October 2020. In Argentina, the mandatory and strict lockdown policies were announced by national government on March 15 [53] and remain active at the time of data collection. This study was approved by the Ethics Committee of the Siglo 21 University (Argentina) and performed in accordance with Helsinki Declaration for studies in humans. Verbal consent was obtained from all participants before completing the questionnaires and no compensation was offered for participation. The verbal informed consent was also approved by the Ethics Committee of the Siglo 21 University.

Data analysis was conducted using SPSS 20.0. A one-way MANOVA was applied to examine differences in mental health outcomes according to work conditions, income loss and self-reported financial problems. Post-hoc comparisons were performed using Bonferroni test, and when assumption of homogeneity of variance was not met, Dunnet’s T3 test were applied. In the case of continuous variables, correlations were calculated using Pearson’s r statistic.

## Results

### Change in working conditions

The MANOVA indicated a statistically significant effect, Wilks λ = 0.96, *F* (5,1006) = 2.51, *p* < 0.001, η_p_^2^ = 0.012. Specifically, univariate ANOVA revealed significant differences in cynicism, *F* (3,1014) = 4.97, *p* = 0.002, η_p_^2^ = 0.015; and life satisfaction, *F* (3,1014) = 5.80, *p* < 0.001, η_p_^2^ = 0.017. In the former case, post hoc analysis showed that people working from home with flexible working hours (*M* = 7.17, *SD* = 5.16) had lower levels of cynicism than those who were working from home with fixed working hours (*M* = 9.36, *SD* = 5.81), those who continued to work at the usual workplace (*M* = 8.74, *SD* = 5.76) and those who were not working because of the lockdown (*M* = 8.50, *SD* = 5.58). In turn, those who work from home with fixed schedules were more cynical than those who continued to work in their usual workplace. With regard to life satisfaction, results showed that people who were not working due to the lockdown reported lower scores (*M* = 20.65, *SD* = 5.33) on life satisfaction than those who were working at the usual workplace (*M* = 22.19, *SD* = 5.04) and those who were working from home with a flexible schedule (*M* = 21.77, *SD* = 4.48). No significant differences were found in exhaustion, *F* (3,1011) = 2.10, *p* = 0.098; anxiety, *F* (3,1011) = 0.15, *p* = 0.927; and depression, *F* (3,1011) = 0.91, *p* = 0.435.

### Financial hardships

#### Income loss

A significant effect of income loss was observed, Wilks λ = 0.96, *F* (5,1007) = 1.51, *p* = 0.043, η_p_^2^ = 0.007. Specifically, univariate ANOVA showed significant differences in life satisfaction, *F* (5,1011) = 2.72, *p* = 0.019, η_p_^2^ = 0.013. Workers with income loss ranging from 81 to 100% report lower life satisfaction (*M* = 20.46, *SD* = 5.38) than those whose income were not affected (*M* = 21.86, *SD* = 4.85).

#### Self-reported financial problems

Results indicated a significant effect of self-reported financial problems, Wilks λ = 0.96, *F* (8,1039) = 4.64, *p* < 0.001, η_p_^2^ = 0.035. Univariate ANOVA indicated differences in cynicism, *F* (1,1045) = 4.52, *p* = 0.034, η_p_^2^ = 0.004; depression, *F* (1,1045) = 10.53, *p* < 0.001, η_p_^2^ = 0.010; anxiety, *F* (1,1045) = 6.87, *p* = 0.009, η_p_^2^ = 0.007; FWC, *F* (1,1045) = 7.43, *p* = 0.007, η_p_^2^ = 0.007; psychological detachment from work, *F* (1,1045) = 7.86, *p* = 0.005, η_p_^2^ = 0.007; and life satisfaction, *F* (1,1045) = 23.92, *p* < 0.001, η_p_^2^ = 0.022. As shown in Table 1, workers who reported financial problems displayed higher scores on cynicism, anxiety, depression and WFC, and lower scores on psychological detachment from work and life satisfaction.

**Table 1:** Mean-group differences in mental health outcomes according to self-reported financial problems (yes/no)

Mental health outcomes	Yes	No
	M (SD)	M (SD)
Cynicism	8.83 (5.65)	8.09 (5.57)
Depression	6.65 (6.16)	5.36 (5.54)
Anxiety	5.93 (5.99)	4.99 (5.60)
Family-work conflict (FWC)	5.85 (3.54)	5.26 (3.34)
Psychological detachment form work	18.19 (5.50)	19.13 (5.25)
Life satisfaction	20.60 (5.29)	22.13 (4.73)

The chi-square test for independence showed that working conditions and self-reported financial problems were related each other, χ^2^(3) = 33.71, *p* < 0.001. People who were not working because of the lockdown reported financial problems more frequently.

### Psychological detachment from work

Psychological detachment from work correlated positively with life satisfaction (*r* = 0.24, *p* < 0.001) and negatively with exhaustion (*r* =  − 0.20, *p* < 0.001), cynicism (*r* =  − 0.08, *p* < 0.01), anxiety (*r* =  − 0.26, *p* < 0.001) and depression (*r* =  − 0.19, *p* < 0.001).

### Work-family conflict (WFC) and family-work conflict (FWC)

WFC was positively related to exhaustion (*r* = 0.37, *p* < 0.001), cynicism (*r* = 0.25, *p* < 0.001), anxiety (*r* = 0.26, *p* < 0.001), and depression (*r* = 0.23, *p* < 0.001), and negatively related to life satisfaction (*r* =  − 0.17, *p* < 0.001). Similarly, FWC was positively related to exhaustion (*r* = 0.24, *p* < 0.001), cynicism (*r* = 0.34, *p* < 0.001), anxiety (*r* = 0.31, *p* < 0.001) and depression (*r* = 0.32, *p* < 0.001), and negatively related to life satisfaction (*r* =  − 0.23, *p* < 0.001).

## Discussion

The COVID-19 pandemic has drastically changed people’ live and work, causing a devastating impact on economies and employment around world. Despite progress in developing vaccines against the virus and their increasing administration to the population, the pandemic is far from over: many countries are now struggling with a resurgence of cases [54, 55]. Thus, countries need to continue exercising lockdown and social distancing to prevent further escalation of the virus and, consequently, the changes in working conditions and income caused by the pandemic are likely to extend over time. Examining the long-term impact of these changes on workers’ mental health is therefore paramount to provide evidence-based recommendations to organisations, employers and governments to develop interventions that mitigate the negative outcomes. Of note, Argentina had one of the world’s longest lockdown [53], thus offering a unique opportunity to evaluate the long-term effect of changes in working conditions and income loss during the pandemic on workers’ mental health.

Our findings indicate that people working from home under fixed schedules (e.g., 9 am to 5 pm) and unemployed reported higher cynicism. A recent study on people’s experiences of home-based telework during the pandemic [33] found that people feel their work less enjoyable and stimulating than it used to be. Working in the physical workspace provides employees with variation in the workday, in the form of social interactions with co-workers, such as going for lunch and having idle conversations during breaks [33]. Such situations are missing in home-based telework which may lead to a monotonous workday, resulting in reduced work motivation (i.e., higher cynicism), particularly for employees working from home under fixed schedules. Our results also showed that people working from home under fixed schedules reported less life satisfaction. As pointed by Almer et al. [56], employees with standard working hour arrangement are less able to structure the workday to accommodate it to their personal needs and balance work and family demands. Consequently, they are more likely to experience work-family conflict and burnout [56, 57] which may in turn influence their life satisfaction [17]. As noted by Cho [7], during COVID-19 lockdown all family members are forced to stay at home all the times, increasing workers’ non-work demands (e.g., childcare, assisting children’s home-based learning, household chores, running errands for the elderly family members who are advised to stay at home). Employees who work from home under fixed schedules may perceive less personal control over the timing and process of work and feel less able to integrate work and family duties, experiencing higher psychological distress [58]. In the case of unemployed, one might argue that because of the unemployment crisis and the decrease in the labor supply, people are less motivated and willing to seek for a job [59]. In addition, unemployed were found to show lower life satisfaction. Work is central to adults’ lives; it not only provides income and a way of satisfying material needs, but also imposes a time structure, enables social relationships, and provides status, sense of self-fulfillment and identity [60, 61]. Not surprisingly, then, job loss can have a negative impact on a person’s life satisfaction, as we found in our study.

Regarding financial hardships, we found that participants with heavy income loss (i.e., 80–100%) during the pandemic reported lower life satisfaction; self-reported financial problems were associated with higher anxiety, depression, burnout, FWC, and with lower psychological detachment from work and life satisfaction. These findings are in line with the large body of pre-pandemic literature [62–66] and with recent COVID-19-related data evidencing the adverse mental health outcomes of the financial burden related to the outbreak [34–37, 67]. For example, Li et al. [34] found that Chinese workers whose income was heavily affected by COVID-19 had higher risk for developing mental health problems such as anxiety and depression. Ruengorn et al. [35] examined income loss and self-reported financial problems related to the pandemic in a national sample of Tai workers. They found that both factors but particularly self-reported financial problems were associated with anxiety, depression and perceived stress, suggesting that the subjective perception of financial strain has more detrimental effects on mental health than the objective indicators (i.e., income loss). In line with this, we found that self-reported financial problems were substantially more related with poor mental health indicators than objective income loss. Importantly, 56.1% of the participants in our sample reported financial problems, and those who became unemployed during the pandemic indicated more financial problems.

Finally, psychological detachment from work, WFC and FWC were unsurprisingly associated with workers’ mental health. Specifically, mental disengagement from work during off-job time was positively related to life satisfaction and negatively correlated to anxiety, depression and burnout symptoms. Conversely, WFC was found to positively correlate with anxiety, depression and burnout, and negatively correlate with life satisfaction, and a similar pattern of results was found for FWC. These findings are consistent with cumulative research on the role of psychological detachment from work and work-family interface [11, 17, 18] and suggest that these factors are also relevant to explain workers’ well-being and mental health during the pandemic.

### Practical implications

Collectively, findings of this study indicate that changes in working conditions and financial hardship as a result of COVID-19 lockdown restrictions have a significant impact on workers’ mental health. As mentioned, those who became unemployed and who shifted to home-based telework with fixed schedules reported impaired mental health. In the latter case, the adoption of flextime could be helpful as it has demonstrated beneficial outcomes for employee well-being [68]. In support of this, our results showed that employees working from home with flexible schedules during the pandemic reported better mental health than those who were working under fixed schedules. On the other hand, creating meaningful interventions to assist newly unemployed may be more challenging because of the diverse contextual and personal factors that characterize this new population [13]. Past research has shown that social support, mastery and perceived control may buffer the negative impact of unemployment on mental health across all ages [69, 70]. Thus, interventions aimed at promoting active coping through online job search workshops that offer instruction to improve job-hunting skills, and career counseling to explore different options of re-employment could be useful to increase job-seeking efforts and perceived control over the situation. Governments can reduce the adverse effects of unemployment through labor market re-employment programs as well as by providing financial aid to companies (e.g., direct financial assistance and facilities to comply with tax obligations, or temporarily reducing or even eliminating them –especially to contact-sensitive sectors which are exposed to considerable vulnerability), in order to alleviate the impact of COVID-19 economic crisis, avoid closures and preserve employment as far as possible. Furthermore, increasing social support through community-based interventions may reduce feelings of distress through the increased availability of coping resources and the reappraisal of the unemployment situation as less stressful [71]. Finally, governments may also contribute to mitigate the negative impact of income loss and financial problems through directly income support and other non-economic measures such as transport facilities, access to healthcare systems, and supply of good and services such as electricity, food, and water [72], especially for the unemployed who are the most affected. It should be noted that some of these recommendations have been already implemented in many countries in response to the COVID-19 first wave [73]. Since countries are tightening restrictions in the face of new waves of the disease –in many cases returning to lockdown and closure of business activities [54, 55], our results point the need to maintain and strengthen support measures to protect greater deterioration of workers’ mental health.

Finally, organisations and employers can also protect workers’ mental health during the pandemic by facilitating mental disengagement from work and reduce the potential for WFC and FWC through work-from-home policies. As stated, the pandemic is far from over and many countries are currently facing a real threat of COVID-19 resurgence. It is thus likely that mandated working from home will continue to some degree in the foreseeable future for millions of workers; organizations can help to protect employees’ mental health through interventions that consider work-home boundary management support, role clarity, workload, performance indicators, technical support, facilitation of co-worker networking and training for managers [74]. For instance, organisations can actively support employees in the designing of the work-family interface through skill-related negotiation training programs for dual-earner couples working from home with children, in order to help them create boundaries and structure cross-boundaries between work and family roles in a manner that enables the integration of the different roles and the reduction of WFC and FWC. They can also facilitate boundary management through clear communication about expectations of working hours to prevent employees feeling as though they are ‘on call 24/7’. Previous studies [75] have shown that employees avoid work-related communication (e.g., texting, making phone calls) after workhours when they perceive a strong organisational norm to do so. Accordingly, education and training of managers on how to set and maintain clear boundaries around the use of information and communication technologies (ICT) for work purposes during non-work time, could facilitate the creation of prescriptive norms about ICT-related use after workhours and formally develop boundaries between work and family. Finally, it has been consistently shown that women working from home are more likely to suffer negative mental health outcomes than men during the COVID-19 pandemic [29, 31, 76]. Thus, working-from-home policies need to address gender inequities to ensure that they meet the nuanced needs of different employees, especially for working mothers who experience a high burden as a result of work and home responsibilities. This burden can be eased by increasing support for childcare and home schooling, including nonfinancial assistance such as training in educational content delivery [77].

### Suggestions for future research

The current study has several limitations that should be acknowledged. Firstly, we examined the impact of changes in working conditions and financial hardships on workers’ mental health various months after the beginning of COVID-19 lockdown; however, our study was cross-sectional. Thus, longitudinal data are necessary to better understand the long-term impact of such changes on workers. For instance, in a 1-month follow up study after the COVID-19 outbreak Hertz-Palmor et al. [67] found that worsening income loss was associated with an increase in depressive symptoms over time. Further longitudinal research using longer time-lags would be valuable to gain deeper insight into the long-term effects of the COVID-19 associated changes on workers’ mental health. Secondly, there are a number of potential variables not addressed in the present study that are likely to influence the impact of changes in working conditions on mental health of workers during the COVID-19 crisis. For example, unemployment may be experienced differently by workers depending on many external circumstances such as financial condition, probability of re-employment following the pandemic, family composition, and living conditions [13]. Moreover, working from home is likely to influence workers’ mental health depending on various factors such as the number of home responsibilities and individuals’ work-home segmentation preferences. It is conceivable that workers who have children 24/7 at home during the pandemic are more susceptible to disruptions while working from home. These disruptions could undermine perceived self-control over work, especially for workers with high segmentation preferences, increasing feelings of stress. Finally, several socio-demographic factors, such as female gender, younger age (≤ 40 years), lower educational level, divorced/widowed status, and the presence of chronic diseases and a history of medical/ psychiatric illnesses, have been shown to be associated with greater risk for developing mental health problems during COVID-19 pandemic [34, 78, 79]. Addressing the influence of these variables and their interaction with changes in working conditions and income loss associated to the COVID-19, will enable for a better understanding of the specific conditions under these changes are more likely to affect workers’ mental health and identifying subgroups at greater risk. Lastly, it would be valuable in future research to include other occupational groups not addressed in the present study, such as underemployed or workers from informal economies, in order to expand results herein.

## Conclusions

The current COVID-19 disease poses a physical threat to human lives, prompting countries to adopt social distancing and lockdown measures to contain the spread of the virus. Although these measures were introduced rapidly at the beginning of the pandemic, they likely will remain for some time in view of the new waves, which calls for studies examining how these changes affect people’s work experience and incomes and their influence on mental health in the long-term. Our results indicate that working from home under fixed schedules and unemployment impact negatively on mental health. Income loss and particularly self-reported financial problems were also associated with mental health problems, in agreement with the growing literature on economic burden and mental health during the COVID‐19 pandemic. Countries’ policies should focus on supporting workers facing economic problems and unemployment to ameliorate the negative impact on mental health; organisations can also protect employees’ mental health by actively encouraging psychological detachment from work and by help managing work-family interface. Further longitudinal studies are needed to more thoroughly assess the long-term impact of the COVID-19-related changes in work and economic turndown on mental health issues.

## Data Availability

The datasets used and/or analysed during the current study are available from the corresponding author on reasonable request.

## Abbreviations

COVID-19: 2019 Novel Coronavirus
WFC: Work-family conflict
FWC: Family-work conflict
REQ: Recovery Experience Questionnaire
SWING: Survey Work-Home Interaction-Nijmegen
MBI-GS: Maslach Burnout Inventory-General Survey
PHQ-9: Patient Health Questionnaire-9 items
GAD-7: Generalised Anxiety Disorder Scale-7 items
SWLS: Satisfaction with Life Scale
